# Ultra-Processed Food and Its Impact on Bone Health and Joint Diseases: A Scoping Review

**DOI:** 10.3390/nu17071188

**Published:** 2025-03-28

**Authors:** Jacopo Ciaffi, Luana Mancarella, Claudio Ripamonti, Andrea D’Amuri, Veronica Brusi, Federica Pignatti, Lucia Lisi, Francesco Ursini

**Affiliations:** 1 Medicine & Rheumatology Unit, IRCCS Istituto Ortopedico Rizzoli, 40136 Bologna, Italy; luana.mancarella@ior.it (L.M.); claudio.ripamonti@ior.it (C.R.); veronica.brusi@ior.it (V.B.); federica.pignatti@ior.it (F.P.); lucia.lisi@ior.it (L.L.); francesco.ursini2@unibo.it (F.U.); 2 Department of Biomedical and Neuromotor Sciences (DIBINEM), Alma Mater Studiorum University of Bologna, 40123 Bologna, Italy; 3 General Medicine Unit, Medical Department, ASST Mantova, Ospedale Carlo Poma, 46100 Mantova, Italy; dmrndr@unife.it

**Keywords:** ultra-processed, food, osteoporosis, bone, osteoarthritis, rheumatoid arthritis, gout

## Abstract

**Background/Objectives**: This scoping review explores the relationship between ultra-processed food (UPF), bone health, and joint diseases, focusing on its potential impact on bone mineral density (BMD), osteoporosis, osteoarthritis, and inflammatory arthritis, including rheumatoid arthritis (RA), gout, and spondyloarthritis. **Methods**: A search strategy was developed using key terms such as “ultra-processed food” and related terms like “fast food,” alongside various definitions of bone health impairment, chronic degenerative joint diseases, and inflammatory arthritis. **Results**: A total of 19 studies were included: 12 on bone health, 3 on osteoarthritis, and 4 on inflammatory arthritis. Preclinical studies showed that UPF consumption negatively affects bone structure and strength. In studies on children and adults, four investigations (2013–2017) found no association between fast food intake and BMD. However, more recent large-scale cross-sectional studies linked higher UPF consumption to lower BMD, increased osteoporosis risk, and greater prevalence of osteopenia, particularly in postmenopausal women. UPF intake was associated with knee osteoarthritis risk, with evidence suggesting an interaction with cartilage thickness, though no association was found for hip osteoarthritis. In inflammatory arthritis, UK Biobank data indicated a higher risk of RA and gout in UPF consumers, while a Brazilian study reported worse metabolic profiles in RA patients. No significant differences in UPF intake were found in spondyloarthritis. **Conclusions**: This review highlights relevant considerations about the deleterious role of UPF on bone health and joint diseases, providing additional evidence to suggest healthier dietary patterns to patients and to the general population.

## 1. Introduction

According to the NOVA Food classification system, which categorizes foods based on the extent and purpose of their processing, ultra-processed foods (UPFs) are industrial formulations with little to no whole foods, containing additives, preservatives and artificial substances to enhance taste, texture, appearance and durability [1]. The most commonly consumed UPFs include soft and sweetened beverages, processed bread, refined breakfast cereals, confectionery products, pre-packaged sauces, ready-to-heat meals and processed meat products [2].

UPFs are a highly relevant topic given the steadily increasing sales and consumption trends, not only in Western societies but also in low- and middle-income countries [3]. UPFs are widespread for several reasons: they are convenient, easy to consume on the go, require no preparation or utensils, are highly palatable, and are typically inexpensive [1]. These undesirable characteristics are further reinforced by aggressive and sophisticated marketing strategies that shape social norms, disproportionately affecting vulnerable groups, particularly children [4]. Nutritionally, UPFs are high in refined sugars, saturated fats, sodium and additives, yet lacking in essential nutrients such as fiber, protein, and key micronutrients including potassium, magnesium, vitamin C, vitamin D, zinc, phosphorus, vitamin B12, niacin and antioxidants [5,6].

These nutritional deficits are strongly linked to poor health outcomes. As energy-dense foods, UPFs contribute to excessive calorie intake and are associated with a 39% higher risk of obesity, a 79% increased risk of metabolic syndrome, and up to a 31% greater risk of diabetes [7,8]. Furthermore, diets rich in UPFs are linked to an elevated risk of coronary artery disease, stroke, cardiovascular mortality and all-cause mortality [9,10,11].

The health risks extend beyond metabolic disorders. UPF consumption has been associated with an increased risk of cancer, including an 11% higher risk of breast tumors, a 30% greater risk of colorectal tumors and a 49% increased risk of pancreatic cancer [12]. Moreover, as scientific research in this field advances, it continues to reveal further health implications. UPF consumption has been linked to cognitive decline, psychiatric disorders such as anxiety and depression, inflammatory bowel disease and demyelinating diseases such as multiple sclerosis [10,13,14,15].

Dietary patterns have long been recognized as key determinants of bone health and osteoporosis risk. Nutrient-rich diets, such as the Mediterranean diet, have been associated with higher bone mineral density (BMD) and lower fracture risk, likely due to their high content of calcium, vitamin D, polyphenols, and omega-3 fatty acids [16,17]. Conversely, Western dietary patterns, characterized by a high intake of refined carbohydrates, saturated fats, and processed foods, have been linked to lower BMD and an increased risk of osteoporosis [18,19]. Most research in this area has focused on specific nutrients, such as calcium, vitamin D, and protein, or on overall dietary patterns rather than on the role of UPF consumption as a distinct risk factor for bone fragility [20]. Given the rising global consumption of UPFs and their well-documented impact on metabolic and inflammatory pathways, investigating their effects on bone health, osteoporosis risk, and joint diseases is of increasing clinical importance [21].

## 2. Materials and Methods

This scoping review was conducted in accordance with the methodological guidelines of the Joanna Briggs Institute [22]. We used the Preferred Reporting Items for Systematic Reviews and Meta-Analysis extension for Scoping Reviews (PRISMA-ScR) checklist to guide the structured reporting of the review (Supplementary File) [23]. The protocol was registered in the Open Science Framework (OSF) Registries (https://doi.org/10.17605/OSF.IO/DEXGB) accessed on 14 February 2025. In line with scoping review’s methodology, no critical appraisal or risk of bias assessment was performed in the included studies [22].

### 2.1. Eligibility Criteria and Study Selection

The Population, Concept, and Context (PCC) framework was used to formulate the research question and define the inclusion criteria. Studies were included if they met the following criteria:

- **Population:**
  ○ Preclinical studies: mice or rat models.
  ○ Human studies: children or adults.
- **Concept:**
  ○ Preclinical models: effects of UPF on bone health, bone growth, histomorphometric properties.
  ○ Clinical studies: impact of UPF on BMD, osteoporosis, osteoarthritis (OA) and inflammatory arthritis incidence; influence of UPF consumption on patients already diagnosed with chronic joint diseases, such as OA and inflammatory arthritis.
- **Context:**
  ○ Preclinical studies: animal models in which UPF-fed mice or rats underwent bone health assessments.
  ○ Bone health and disease risk: studies evaluating the association between UPF consumption and the risk of developing osteoporosis, low BMD, fractures, or inflammatory arthritis.
  ○ Impact on existing conditions: Studies investigating the effects of UPF intake on disease activity, progression, and related metabolic or inflammatory outcomes in patients with established chronic joint diseases.

We included randomized controlled trials (RCTs), quasi-RCTs, prospective and retrospective cohort studies, case–control studies, cross-sectional studies, and qualitative reports. Conference proceedings were also considered.

### 2.2. Search Strategy

After having registered the protocol on OSF, we conducted a comprehensive literature search in MedLine (via PubMed), Web of Science (WOS), and Embase, up to 14 February 2025. The search strategy included key terms related to UPF, bone health, and chronic joint diseases. Specifically, we used terms such as “ultra-processed food,” “highly processed food,” and “fast food” to identify dietary exposures. For the assessment of the outcomes, we included terms related to bone health (e.g., bone mineral density, osteoporosis, osteopenia, bone growth, bone health, bone diseases, fracture, bone loss) and chronic joint diseases (e.g., osteoarthritis, rheumatoid arthritis, psoriatic arthritis, spondyloarthritis, ankylosing spondylitis, gout). To ensure full transparency and reproducibility, the complete search strategy for each database is provided as a Supplementary File.

We also performed manual searches using relevant keywords and screened the references of included articles to find additional sources. The search strategy was developed and conducted by two independent authors (J.C. and L.M.) and supervised by a senior investigator (F.U.). No restrictions were applied on publication date.

### 2.3. Study Selection and Data Charting

After the removal of duplicate records, two reviewers (J.C. and L.M.) independently screened all titles and abstracts of the retrieved articles. Full-text assessment was performed for potentially eligible studies. Any discrepancies during the study selection process were resolved through discussion, and a senior investigator (F.U.) was consulted when consensus could not be reached.

For each included study, the following information was extracted: first author, year of publication, country, study design, sample size, study population (preclinical models or human participants), exposure assessment (UPF consumption), comparator groups (if applicable), outcomes assessed (e.g., bone mineral density, osteoporosis, fractures, osteoarthritis, inflammatory arthritis), and key findings related to UPF and musculoskeletal health.

### 2.4. Synthesis of Results

We conducted a narrative synthesis of the extracted data, structured into five main sections based on study populations and disease areas:

**Preclinical studies on bone health**
  - This section summarizes findings from animal models assessing the impact of UPF consumption on bone health.
  - Key outcomes include changes in bone biomechanical properties, histomorphometric parameters, bone growth, and mineralization in rodents fed with UPF diets.
**UPF and bone health in children and adolescents**
  - This section compiles evidence from studies evaluating the relationship between UPF consumption and BMD or bone health markers in pediatric and adolescent populations.
  - Findings include differences in BMD, bone turnover markers, and skeletal growth patterns in children with high UPF intake.
**UPF and bone health in adults**
  - This section synthesizes clinical studies exploring the association between UPF intake and bone health in adults.
  - Key outcomes include the impact of UPF on osteopenia, osteoporosis, fracture risk, and changes in BMD in different adult populations.
**UPF and osteoarthritis**
  - This section summarizes studies investigating the link between UPF consumption and the risk or progression of OA.
  - Outcomes include the prevalence and severity of OA, cartilage integrity, pain levels, and functional impairment in individuals with high UPF consumption.
**UPF and inflammatory arthritis**
  - This section reviews evidence on UPF intake and the risk or progression of inflammatory arthritis, including rheumatoid arthritis (RA), spondyloarthritis (SpA), and gout.
  - Findings cover the influence of UPF on disease incidence, inflammation markers, symptom severity, disease progression, and metabolic comorbidities in patients with inflammatory arthritis.

## 3. Results

The database search yielded a total of 119 studies (PubMed: 24, WOS: 50, Embase: 45). After removing 40 duplicates, 79 records remained for screening. Following title and abstract screening, 60 studies were excluded, leaving 19 full-text articles that met the inclusion criteria and were included in the qualitative synthesis (Table 1).

Of the 19 included studies, 12 focused on bone health, with 3 preclinical and 9 clinical studies, while 7 investigated chronic joint diseases. Additionally, four studies were conference proceedings. The studies were published between 2013 and 2025 and originated from the United States (5), China (4), Korea (3), Israel (2), Brazil (1), the United Kingdom (1), Portugal (1), Japan (1) and France (1).

**Figure 1:**
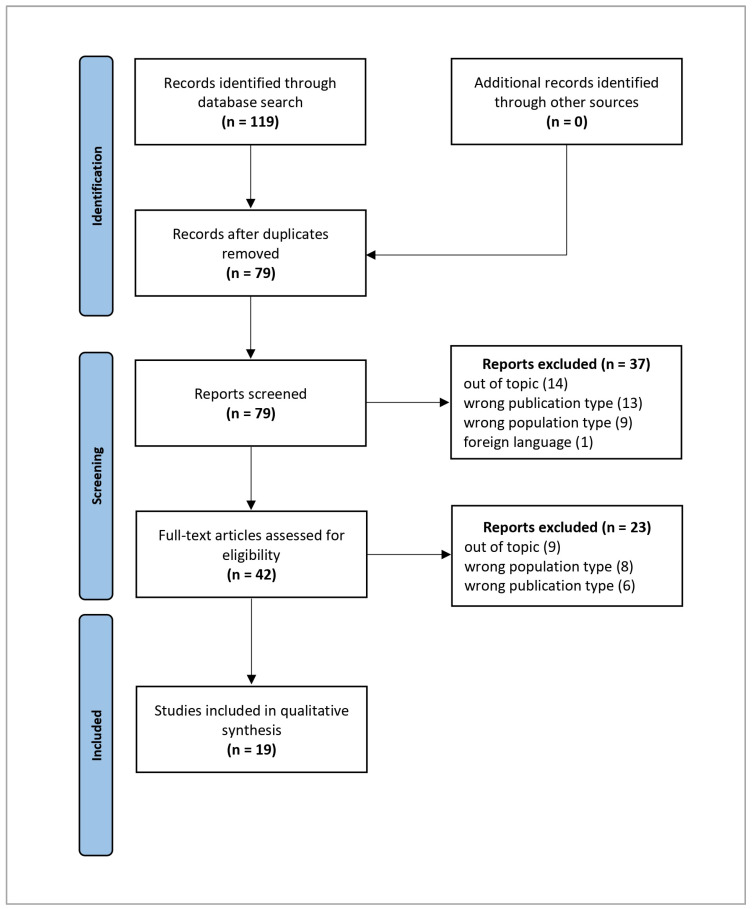
PRISMA 2020 flow diagram. Adapted From: Page MJ, McKenzie JE, Bossuyt PM, Boutron I, Hoffmann TC, Mulrow CD, et al. The PRISMA 2020 statement: an updated guideline for reporting systematic reviews [43].

A flowchart illustrating the study selection process is presented in Figure 1.

### 3.1. Preclinical Studies on Bone Health

Three preclinical studies have investigated the impact of UPF consumption on bone health, demonstrating detrimental effects on BMD, microarchitecture, and mechanical properties [31,36,40].

A study in young female mice found that UPF consumption resulted in a significant reduction in bone quality [40]. Micro-CT scans revealed impaired bone morphology and biomechanical testing showed inferior bone strength. Additionally, marrow adiposity was significantly increased, suggesting that UPF consumption alters bone remodeling processes and could predispose individuals to osteoporosis [40].

Similarly, in young rats, a high-fat, high-sugar UPF diet led to severe skeletal impairments, including growth retardation, lesions in the tibial growth plates, and a significant decrease in BMD [36]. Structural deterioration was observed in both trabecular and cortical bone. RNA sequencing revealed disruptions in extracellular matrix formation and mineralization processes. These findings suggest that UPF exposure during growth may lead to long-term skeletal fragility and increased fracture risk [36].

A third study examined the effects of a calorie-dense UPF “cafeteria” diet in male rats, investigating whether resistance exercise (ladder climbing) could counteract the diet’s effects [31]. The results showed that UPF consumption led to a significant increase in adipose tissue, a reduction in BMD, and impaired biomechanical properties such as stiffness and maximum load capacity. Notably, exercise did not prevent the deterioration in bone quality, suggesting that dietary interventions are necessary to mitigate the negative skeletal effects of UPF consumption.

Collectively, these preclinical studies highlight the adverse effects of UPF on bone growth and integrity, demonstrating that UPF consumption negatively affects bone mass, structure, and strength, potentially leading to an increased risk of osteoporosis and fractures. Furthermore, exercise alone may not be sufficient to counteract these effects, and dietary modifications are needed to maintain bone health.

### 3.2. UPF and Bone Health in Children and Adolescents

We found four studies investigating the impact of UPF and fast food consumption on bone health in children and adolescents [26,28,33,37].

Lim et al. analysed the impact of fast food consumption on BMD in college students, finding a significant association between frequent fast food intake and lower BMD in both males and females [26]. The study also revealed nutrient imbalances, with excess sodium and protein intake coupled with deficiencies in vitamins A and C, suggesting that the poor micronutrient profile of UPFs may contribute to impaired bone metabolism. A UPF-rich diet may thus interfere with bone mineralization during young adulthood, a critical period for achieving peak bone mass [26].

The role of the food environment in childhood bone health was assessed by Vogel et al. in a study of young children from the Southampton Women’s Survey [37]. Greater exposure to fast food outlets in residential areas was associated with lower BMD and bone mineral content at birth and during early childhood. Specifically, each additional fast food outlet near a child’s home correlated with a 0.23 standard deviation decrease in BMD. In contrast, greater access to healthy food stores was associated with higher BMD at ages 4 and 6, suggesting that the availability of fast food influences long-term bone health outcomes from infancy onward [37].

In a longitudinal study of Portuguese adolescents, Monjardino et al. examined how dietary patterns affected bone accrual from ages 13 to 17 [28]. While no significant associations were found between the fast food dietary pattern and mean BMD at 13 years, girls who followed a diet characterized by lower food intake, including lower consumption of nutrient-dense foods, showed significantly lower BMD accrual over time (adjusted coefficient: −0.451 mg/cm^2^/year). The findings suggest that nutrient-poor diets, which often include processed foods, may contribute to reduced bone mineralization during adolescence [28].

In a study of Korean adolescents, Shin et al. evaluated the impact of different dietary patterns on lumbar spine and femoral BMD [33]. No direct link was found between a fast-food-heavy diet and lower BMD, but the findings suggested a trend toward poorer bone health outcomes in those consuming higher amounts of UPF. Conversely, adolescents following a milk and cereal-rich dietary pattern had a 64% lower likelihood of low BMD, reinforcing the importance of whole, nutrient-rich foods in skeletal development [33].

These four studies highlight the potential risks of UPF consumption for bone health in children and adolescents. Frequent fast food intake and greater exposure to fast food outlets are associated with lower BMD, impaired bone accrual, and reduced bone strength.

### 3.3. UPF and Bone Health in Adults

Our search strategy identified five studies, including four full papers and one conference poster, that explored the association between UPF and bone health outcomes in adults, particularly in relation to BMD, osteoporosis, and fracture risk [25,27,30,32,38].

A large cross-sectional analysis by Greatorex Brooks et al. used NHANES data to examine UPF consumption in U.S. adults aged 50 and older [25]. The study found that higher UPF intake was significantly associated with an increased prevalence of osteopenia and osteoporosis, particularly in women. Individuals in the highest quintile of UPF consumption had a 52% greater likelihood of osteoporosis compared to those in the lowest quintile. Furthermore, each 1% increase in UPF intake correlated with a 1.9% increase in self-reported fractures among women, suggesting a direct link between UPF consumption and skeletal fragility [25].

Similarly, Wang et al. conducted a NHANES-based study and found that individuals whose diet consisted of more than 57.5% UPF had significantly lower BMD at the femoral neck and total femur, which are considered key sites for osteoporotic fractures [38]. High intake of UPF was associated with 78.9% increased odds of developing osteoporosis. The study also highlighted that physical activity could partially mitigate the negative impact of UPF on bone health, thus highlighting the role of lifestyle factors in influencing the skeletal deterioration related to UPF intake [38].

While these two studies demonstrated clear negative effects of UPF on bone health, Shin et al. examined dietary patterns in Korean adults and found no direct association between fast food intake and BMD [32]. However, the authors reported that a fruit, milk, and whole-grain-based dietary pattern was positively correlated with BMD, suggesting that a diet rich in essential nutrients may counteract bone loss. This highlights the complexity of dietary influences on bone health and suggests that the nutrient density of a diet may be more predictive of BMD than the presence of UPF alone [32].

Supporting the detrimental impact of UPF, a conference poster presented by Noel et al. investigated UPF consumption and osteoporosis in Puerto Rican adults aged 47–79 years [30]. The study found that higher UPF intake was significantly associated with increased odds of osteoporosis, particularly at the femoral neck and lumbar spine. Even after adjusting for key confounders like age, sex, BMI, and lifestyle habits, the association remained significant, reinforcing the idea that UPF consumption is an independent risk factor for osteoporosis [30].

Finally, Mangano et al. examined dietary protein sources and musculoskeletal health in the Framingham Third Generation cohort [27]. Among the different dietary patterns identified, one characterized by high fast food and full-fat dairy intake showed no significant association with BMD. However, higher total protein intake was linked to improved muscle mass and strength, indicating that protein consumption may help preserve musculoskeletal health, even in the presence of some UPF intake [27].

Taken together, these studies suggest a strong association between high UPF consumption and poor bone health outcomes in adults. Higher UPF intake is linked to lower BMD, increased osteoporosis risk, and greater fracture prevalence, particularly in older populations. However, some findings indicate that nutrient-dense dietary patterns and adequate protein intake may counteract these negative effects.

### 3.4. UPF and Osteoarthritis

We identified three studies, including two conference posters, that examined the relationship between UPF consumption and OA [24,34,39]. These studies focused on cartilage structure, pain severity, and OA risk in individuals with high UPF intake.

Wei et al. conducted a large prospective cohort study using data from the UK Biobank (n = 163,987 participants) to assess the impact of UPF consumption on hip and knee OA risk [39]. The study found that individuals in the highest quartile of UPF consumption had a 10% increased risk of developing knee OA compared to those in the lowest quartile, while no significant association was observed for hip OA. Additionally, replacing 20% of UPF intake with unprocessed or minimally processed food was linked to a 6% lower risk of knee OA, thus suggesting that dietary modifications could reduce the risk of developing OA [39].

A conference abstract by Akkaya et al. used Osteoarthritis Initiative (OAI) data to examine the association between UPF consumption and knee cartilage thickness in 4330 individuals [24]. The results showed a significant interaction between UPF intake, cartilage region, and sex. In women, higher UPF intake was associated with thinner cartilage in the medial tibia, medial femur, and lateral femur. In men, the association was weaker and mostly non-significant [24].

Another conference poster by Sims et al. analysed the relationship between UPF consumption and OA-related pain and functional outcomes in 4796 participants from the OAI cohort [34]. The study found that, after adjusting for potential confounders, including BMI, women consuming higher amounts of UPF experienced greater knee OA-related pain, worse activities of daily living, and lower physical performance compared to men [34].

These studies suggest that high UPF consumption could be linked to an increased risk of knee OA, reduced cartilage thickness, and worse pain outcomes, particularly in women. While no significant association was found for hip OA, findings from the UK Biobank and OAI cohorts indicate that reducing UPF intake could be a potential strategy for knee OA prevention and symptom management.

### 3.5. UPF and Inflammatory Arthritis

We identified four studies, including one conference abstract, that investigated the impact of UPF consumption on inflammatory arthritis, specifically RA, SpA, and gout [29,35,41,42]. These studies examined disease risk, symptom severity, and metabolic outcomes in individuals consuming high amounts of UPF.

A retrospective cohort study by Zhao et al. using the UK Biobank (n = 207,012 participants) evaluated the association between UPF consumption and RA incidence [42]. Participants in the highest quintile of UPF consumption had a 17% increased risk of developing RA compared to the lowest quintile. Mediation analyses suggested that inflammation, lipid profile changes, and liver enzyme alterations partially explained the link between UPF intake and RA risk, accounting for 3–15% of the association [42].

Another cross-sectional study by Smaira et al. examined the association between UPF consumption and cardiovascular risk in RA patients [35]. Among 56 individuals with RA, higher UPF intake was significantly associated with worsened metabolic and cardiovascular health, including an increased Framingham cardiovascular risk score and elevated glycated hemoglobin levels. Conversely, patients who consumed more unprocessed or minimally processed foods had a lower cardiovascular risk [35].

A prospective cohort study by Zhang et al. explored the relationship between UPF consumption, genetic predisposition, and gout risk in 181,559 participants from the UK Biobank [41]. The study found that higher UPF intake was associated with a 16% increased risk of developing gout. Additionally, individuals with both high genetic predisposition and high UPF intake had a nearly twofold increased risk of developing gout compared to those with low genetic predisposition and low UPF intake. The study also performed substitution analyses, showing that replacing 20% of daily UPF consumption with unprocessed or minimally processed foods reduced gout risk by 13% [41].

Finally, a conference abstract by Nguyen et al. investigated the dietary profiles of 140 patients with SpA and assessed their intake of UPF and its association with disease activity [29]. The study found no significant differences in UPF consumption between patients with active and inactive SpA [29].

Evidence from the four included studies on inflammatory arthritis suggests that higher UPF consumption is associated with increased risks of RA and gout, while its impact on SpA remains unclear. Moreover, among patients already diagnosed with RA, higher UPF intake was linked to worse metabolic health and increased cardiovascular risk.

## 4. Discussion

Our scoping review identified key insights into the relationship between UPF consumption, bone health, and chronic joint diseases. In the field of bone health and osteoporosis, there is a substantial body of evidence, encompassing both preclinical studies assessing bone microarchitecture and large-scale epidemiological studies evaluating osteoporosis and fracture risk. In contrast, research on UPF and chronic joint diseases remains limited, with only two large prospective cohort studies investigating the association between UPF intake and the risk of developing RA and gout.

This disparity was reflected in our review, as we identified 12 studies focusing on bone health, whereas only 7 studies addressed chronic joint diseases, of which only 4 were full-text articles, with the remaining being conference abstracts or posters. These findings highlight an important gap in the literature and the need for more robust, high-quality studies to explore the impact of UPF on musculoskeletal diseases, particularly in the context of inflammatory arthritis and OA progression.

Our findings were structured into five key research objectives. Preclinical studies consistently showed that UPF consumption deteriorates bone quality, leading to reduced BMD, altered trabecular structure, and impaired biomechanical properties [31,36,40]. These results suggest that early dietary exposure to UPF may have lasting negative effects on skeletal integrity, contributing to weakened bone strength and increased fracture risk. Further research is needed to clarify how inflammation and nutrient imbalances mediate these effects on bone remodeling.

We examined the association between UPF intake and bone health in children and adolescents. Studies linked higher UPF consumption to lower BMD at key skeletal sites, including the lumbar spine and femur [26,28]. Additionally, greater exposure to fast food environments correlated with reduced BMD in children, emphasizing the role of food accessibility in bone development [37]. Since early dietary habits shape peak bone mass and long-term skeletal health, these findings highlight the need for preventive strategies to limit UPF intake in youth and mitigate future skeletal risks [44,45].

In adults, large-scale observational studies found an association between increased UPF intake, lower BMD and increased osteoporosis risk, particularly in postmenopausal women and older individuals [25]. NHANES data confirmed this inverse relationship, raising concerns about UPF-driven skeletal aging [38]. Although the cross-sectional design of some studies limits causal inference, these findings align with evidence on the metabolic and inflammatory effects of UPFs, reinforcing the need for dietary modifications in osteoporosis prevention [46].

Furthermore, our review identified an association between high UPF intake and increased knee OA risk, though no significant effect was observed for hip OA [39]. UPF consumption was also correlated with worse pain, reduced physical function, and thinner knee cartilage, suggesting a role in disease progression via inflammatory and metabolic pathways [24,34]. However, the lack of significant findings for hip OA highlights inconsistencies, emphasizing the need for further research to clarify these associations [24,34].

Finally, we evaluated the impact of UPF consumption on inflammatory arthritis, including RA, SpA, and gout. Higher UPF intake was associated with a 17% increased risk of RA and a 16% increased risk of gout, according to large prospective studies [41,42]. However, findings on SpA were inconclusive, with no significant associations between UPF intake and disease activity. While existing cohort studies provide initial evidence linking UPFs to inflammatory arthritis, further research is needed to establish causality and mechanistic pathways, particularly regarding diet-induced inflammation. The evidence for SpA remains insufficient, underscoring the need for future studies on the role of UPF in disease onset and progression.

The findings of this review highlight the potential public health burden of UPF consumption on musculoskeletal health, reinforcing the need for dietary interventions and policy changes. The widespread availability, aggressive marketing, and affordability of UPFs have led to their increasing consumption across all age groups, including children, adolescents, and older adults, who are particularly vulnerable to bone fragility and joint diseases [47,48]. Given that UPFs are energy-dense but nutrient-poor, their high content of refined sugars, trans fats, sodium, and food additives, combined with their low levels of essential micronutrients such as calcium, vitamin D, magnesium, and antioxidants, may significantly contribute to bone loss, osteoporosis risk, and cartilage degeneration [7,49].

In addition, UPFs have been strongly linked to systemic inflammation, oxidative stress, metabolic syndrome, and obesity, all of which play a key role in osteoporosis, OA, and inflammatory arthritis [50,51]. The association between UPF consumption and an increased risk of RA and gout suggests that dietary modifications may be a potentially modifiable strategy for reducing the disease burden of inflammatory arthritis [52,53]. However, while the connection between UPFs and metabolic diseases such as obesity, diabetes, and cardiovascular disease is well established, their musculoskeletal effects remain underexplored, necessitating further research to determine causal pathways [5,8,54].

While our review highlights important associations between UPF consumption, bone health, and joint diseases, several limitations should be acknowledged. Most included studies were observational, preventing the establishment of causal relationships. Although some studies utilized prospective designs or genetic risk adjustment to strengthen causal inference, the possibility of reverse causation and residual confounding cannot be excluded [38,41]. The extent to which confounding factors were accounted for in the included studies is an important consideration. While 10 of the 16 clinical studies adjusted for confounders, the range and depth of adjustments varied considerably [25,26,27,28,32,33,38,39,41,42]. Most studies controlled for age, sex, BMI, and energy intake, but other key lifestyle and health factors, such as physical activity, smoking, alcohol consumption, socioeconomic status, corticosteroid use, and comorbid inflammatory conditions, were often not included in statistical models. Given the well-established influence of these factors on bone metabolism and joint health, their omission introduces the possibility of residual confounding, which may partially explain inconsistencies across studies.

To improve the quality and reliability of future research, greater efforts should be made to reduce methodological heterogeneity, particularly by incorporating more robust and standardized dietary assessment tools. Well-controlled longitudinal studies and Mendelian randomization approaches may help clarify causal pathways and minimize the risk of reverse causation and unmeasured confounding in this field [55].

Additionally, an important limitation of the included studies is the lack of consistency in how UPF exposure was assessed. There was substantial variability in the methods used to quantify UPF intake, which could contribute to heterogeneity in findings. Most studies relied on self-reported dietary data, often collected through food frequency questionnaires (FFQs) or 24 h dietary recalls, both of which are subject to recall bias and misclassification [56]. The classification of UPFs also varied across studies, with some using established food databases based on NOVA categorization, while others employed broader dietary pattern analyses, which may not fully capture UPF exposure. Moreover, some studies used indirect measures to assess UPF intake, such as fast-food outlet density in a given area, assuming that greater availability translates to higher individual consumption [37]. While such approaches provide valuable insights into environmental influences on diet, they do not directly measure personal dietary intake, making it difficult to draw precise conclusions about the relationship between UPF consumption and musculoskeletal health outcomes. This methodological variability may, at least in part, explain why the association between UPF intake, bone health, and joint diseases was not consistent across all studies. Differences in dietary assessment tools, classification criteria, and exposure measurement could have influenced the observed effect sizes and statistical significance, contributing to variability in findings. Standardizing UPF exposure assessment in future research could help improve comparability and reproducibility of results, ultimately providing stronger evidence on the role of UPFs in musculoskeletal health.

Notwithstanding the abovementioned limitations, from a public health perspective, the findings of our review support the need for nutritional education programs, clearer food labeling, and policies aimed at reducing UPF consumption, particularly among high-risk populations such as postmenopausal women, individuals with OA, and those predisposed to inflammatory arthritis. Strategies such as taxation of UPFs, restrictions on advertising to children, and incentives for healthier food choices could help curb the rising trend of UPF consumption and mitigate its long-term impact on bone and joint health [57]. Future studies should explore whether dietary interventions that reduce UPF intake can effectively slow disease progression and improve musculoskeletal outcomes, offering a cost-effective and accessible approach to musculoskeletal disease prevention.

## 5. Conclusions

The growing body of evidence on UPF consumption and adverse health outcomes highlights its detrimental impact not only on metabolic and cardiovascular diseases but also on bone health and chronic joint diseases. Our review suggests that high UPF intake is associated with reduced BMD, increased osteoporosis risk, and worse clinical outcomes in OA and inflammatory arthritis. However, most of the available evidence comes from observational studies, limiting the ability to draw causal conclusions. While some studies, such as those using prospective cohort designs or genetic risk adjustment, provide stronger evidence, methodological heterogeneity, including variability in dietary assessment methods and incomplete adjustment for confounders, remains a major limitation.

Despite accumulating evidence, research in this field is still in its early stages, particularly regarding the long-term impact of UPFs on musculoskeletal health. Given the global rise in UPF consumption, future studies should focus on well-controlled longitudinal research and intervention trials to determine whether reducing UPF intake could serve as a modifiable strategy to protect bone health and joint integrity. Additionally, standardized methods for UPF exposure assessment and more comprehensive adjustment for confounding factors will be essential for improving study comparability.

From a public health perspective, efforts should prioritize nutritional education and dietary interventions, promoting the consumption of whole, nutrient-dense foods to mitigate the growing burden of musculoskeletal diseases associated with UPFs.

## Figures and Tables

**Table 1:** Characteristics of the included studies.

First Author	Year of Publication	Country of Affiliation of First Author	Publication Type	Study Design	Sample Size	Study Population	Exposure Assessment	Outcomes Assessed	Key Findings
Akkaya Z. [24]	2024	United States	Conference proceeding	Cross-sectional	4330	OA patients from the Osteoarthritis Initiative cohort	Self-reported UPF intake	Cartilage thickness in knee OA	Greater UPF intake was linked to thinner knee cartilage, particularly in women with OA.
Greatorex Brooks E.L. [25]	2025	United States	Full-text article	Cross-sectional	5729	adults from the NHANES cohort	UPF % of total energy intake	Osteoporosis prevalence and BMD	Higher UPF consumption was associated with increased osteoporosis prevalence and lower BMD in NHANES participants.
Lim H.S. [26]	2018	Korea	Full-text article	Cross-sectional	161	college students	Self-reported frequency of fast food consumption	Total body BMD and dietary influence	Frequent fast-food consumption was negatively associated with total body BMD in college students.
Mangano K.M. [27]	2017	United States	Full-text article	Prospective cohort	2986	adults	Dietary protein clusters, including “Fast food and full-fat dairy”	BMD and lean mass association	Higher protein intake, even from processed sources, was associated with greater lean mass but had no clear effect on BMD.
Monjardino T. [28]	2015	Portugal	Full-text article	Prospective cohort	1007	adolescents	Dietary patterns including “Fast food and sweets” cluster	BMD at the lumbar spine and femoral neck	Higher intake of fast food and sweets was associated with lower BMD at the lumbar spine and femoral neck in adolescents.
Nguyen K. [29]	2019	France	Conference proceeding	Cross-sectional	140	SpA patients	Self-reported UPF consumption	Disease activity and quality of life	UPF intake was not significantly different between active and inactive SpA patients, but poor diet was associated with worse quality of life.
Noel S. [30]	2023	United States	Conference proceeding	Cross-sectional	1254	adults	Self-reported UPF intake frequency	Osteoporosis prevalence and BMD	Puerto Rican adults with high UPF intake had lower BMD and a higher prevalence of osteoporosis.
Saito M.K. [31]	2024	Japan	Full-text article	Prospective preclinical	48	rats	Cafeteria diet (highly processed) vs. standard chow	BMD, trabecular structure, and biomechanical properties	Rats fed a cafeteria diet exhibited lower BMD, reduced trabecular bone structure, and weaker biomechanical properties compared to controls.
Shin S. [32]	2015	Korea	Full-text article	Cross-sectional	3573	adults	Dietary patterns categorized, including “Fast food and soda” group	Association between dietary patterns and BMD	A “fast food and soda” dietary pattern was associated with lower BMD in adults.
Shin S. [33]	2013	Korea	Full-text article	Cross-sectional	196	adolescents	Dietary patterns categorized as “Fast food” vs. “Milk and Cereal”	BMD at the lumbar spine, femur, and total body	A diet rich in fast food was linked to lower BMD at multiple skeletal sites in adolescents.
Sims W. [34]	2024	United States	Conference proceeding	Cross-sectional	4796	OA patients from the Osteoarthritis Initiative cohort	UPF % of total energy intake	OA-related pain, physical function, and disability	Higher UPF consumption was associated with worse OA-related pain and poorer physical function.
Smaira F.I. [35]	2020	Brasil	Full-text article	Cross-sectional	56	RA patients	Self-reported UPF intake frequency	Cardiovascular risk factors and metabolic health	RA patients consuming more UPF had worse metabolic profiles and higher cardiovascular risk.
Travinsky-Shmul T. [36]	2021	Israel	Full-text article	Prospective preclinical	36	mice	UPF diet with food additives vs. control diet	Bone strength, mechanical properties, and histological changes	Mice consuming a UPF-based diet had significantly reduced bone strength, altered mechanical properties, and increased histological abnormalities.
Vogel C. [37]	2016	United Kingdom	Full-text article	Ecological	1107	children	Number of fast food outlets near residence	Association between fast food exposure and BMD	Children living in areas with greater fast food availability had lower BMD, suggesting an environmental impact on bone health.
Wang S. [38]	2024	China	Full-text article	Cross-sectional	4912	adults from the NHANES cohort	UPF % of daily caloric intake	BMD at femoral neck and osteoporosis risk	Adults with high UPF intake had lower femoral neck BMD and a significantly greater risk of osteoporosis.
Wei Y. [39]	2024	China	Full-text article	Prospective cohort	163,987	adults	UPF % of total caloric intake	Risk of knee and hip osteoarthritis	High UPF consumption increased the risk of knee osteoarthritis but had no significant effect on hip OA.
Zaretsky J. [40]	2021	Israel	Full-text article	Prospective preclinical	40	rats	UPF-based diet vs. control diet	Bone microarchitecture and endochondral ossification defects	UPF consumption led to impaired bone microarchitecture and disrupted endochondral ossification in rats, suggesting negative effects on bone quality.
Zhang T. [41]	2024	China	Full-text article	Prospective cohort	181,559	adults from the UK biobank study	UPF intake categorized by quartiles	Risk of developing gout and genetic interactions	Higher UPF consumption was associated with an increased risk of gout, particularly in genetically predisposed individuals.
Zhao H. [42]	2024	China	Full-text article	Prospective cohort	207,012	adults from the UK biobank study	UPF % of total dietary intake	Risk of developing rheumatoid arthritis	Individuals with high UPF intake had a 17% increased risk of developing RA.

BMD: bone mineral density; OA: osteoarthritis; RA: rheumatoid arthritis; SpA: spondyloarthritis; UK: United Kingdom; UPF: ultra-processed food.

## Data Availability

No new data were created or analyzed in this study. Data sharing is not applicable to this article.

## Supplementary Materials

The following supporting information can be downloaded at: https://www.mdpi.com/article/10.3390/nu17071188/s1, PRISMA-ScR-checklist and search strategy.

## References

[1] Monteiro C.A., Cannon G., Levy R.B., Moubarac J.-C., Louzada M.L., Rauber F., Khandpur N., Cediel G., Neri D., Martinez-Steele E. (2019). Ultra-Processed Foods: What They Are and How to Identify Them. Public Health Nutr..

[2] Wang L., Du M., Wang K., Khandpur N., Rossato S.L., Drouin-Chartier J.-P., Steele E.M., Giovannucci E., Song M., Zhang F.F. (2022). Association of Ultra-Processed Food Consumption with Colorectal Cancer Risk among Men and Women: Results from Three Prospective US Cohort Studies. BMJ.

[3] Baker P., Machado P., Santos T., Sievert K., Backholer K., Hadjikakou M., Russell C., Huse O., Bell C., Scrinis G. (2020). Ultra-Processed Foods and the Nutrition Transition: Global, Regional and National Trends, Food Systems Transformations and Political Economy Drivers. Obes. Rev..

[4] Mallarino C., Gómez L.F., González-Zapata L., Cadena Y., Parra D.C. (2013). Advertising of Ultra-Processed Foods and Beverages: Children as a Vulnerable Population. Rev. Saude Publica.

[5] Juul F., Vaidean G., Parekh N. (2021). Ultra-Processed Foods and Cardiovascular Diseases: Potential Mechanisms of Action. Adv. Nutr..

[6] Martini D., Godos J., Bonaccio M., Vitaglione P., Grosso G. (2021). Ultra-Processed Foods and Nutritional Dietary Profile: A Meta-Analysis of Nationally Representative Samples. Nutrients.

[7] Pagliai G., Dinu M., Madarena M.P., Bonaccio M., Iacoviello L., Sofi F. (2021). Consumption of Ultra-Processed Foods and Health Status: A Systematic Review and Meta-Analysis. Br. J. Nutr..

[8] Delpino F.M., Figueiredo L.M., Bielemann R.M., da Silva B.G.C., Dos Santos F.S., Mintem G.C., Flores T.R., Arcêncio R.A., Nunes B.P. (2022). Ultra-Processed Food and Risk of Type 2 Diabetes: A Systematic Review and Meta-Analysis of Longitudinal Studies. Int. J. Epidemiol..

[9] Du S., Kim H., Rebholz C.M. (2021). Higher Ultra-Processed Food Consumption Is Associated with Increased Risk of Incident Coronary Artery Disease in the Atherosclerosis Risk in Communities Study. J. Nutr..

[10] Bhave V.M., Oladele C.R., Ament Z., Kijpaisalratana N., Jones A.C., Couch C.A., Patki A., Garcia Guarniz A.-L., Bennett A., Crowe M. (2024). Associations Between Ultra-Processed Food Consumption and Adverse Brain Health Outcomes. Neurology.

[11] Dai S., Wellens J., Yang N., Li D., Wang J., Wang L., Yuan S., He Y., Song P., Munger R. (2024). Ultra-Processed Foods and Human Health: An Umbrella Review and Updated Meta-Analyses of Observational Evidence. Clin. Nutr..

[12] Isaksen I.M., Dankel S.N. (2023). Ultra-Processed Food Consumption and Cancer Risk: A Systematic Review and Meta-Analysis. Clin. Nutr..

[13] Ejtahed H.-S., Mardi P., Hejrani B., Mahdavi F.S., Ghoreshi B., Gohari K., Heidari-Beni M., Qorbani M. (2024). Association between Junk Food Consumption and Mental Health Problems in Adults: A Systematic Review and Meta-Analysis. BMC Psychiatry.

[14] Narula N., Chang N.H., Mohammad D., Wong E.C.L., Ananthakrishnan A.N., Chan S.S.M., Carbonnel F., Meyer A. (2023). Food Processing and Risk of Inflammatory Bowel Disease: A Systematic Review and Meta-Analysis. Clin. Gastroenterol. Hepatol..

[15] Mannino A., Daly A., Dunlop E., Probst Y., Ponsonby A.-L., van der Mei I.A.F., Black L.J., Ausimmune Investigator Group (2023). Higher Consumption of Ultra-Processed Foods and Increased Likelihood of Central Nervous System Demyelination in a Case-Control Study of Australian Adults. Eur. J. Clin. Nutr..

[16] Rizzoli R., Biver E., Bonjour J.-P., Coxam V., Goltzman D., Kanis J.A., Lappe J., Rejnmark L., Sahni S., Weaver C. (2018). Benefits and Safety of Dietary Protein for Bone Health—An Expert Consensus Paper Endorsed by the European Society for Clinical and Economical Aspects of Osteopororosis, Osteoarthritis, and Musculoskeletal Diseases and by the International Osteoporosis Foundation. Osteoporos. Int..

[17] Weaver C. (2017). Nutrition and Bone Health. Oral Dis..

[18] Muñoz-Garach A., García-Fontana B., Muñoz-Torres M. (2020). Nutrients and Dietary Patterns Related to Osteoporosis. Nutrients.

[19] Casey C., Kemp B.J., Cassidy L., Patterson C.C., Tully M.A., Hill A.J., McCance D.R. (2023). The Influence of Diet and Physical Activity on Bone Density of Children Aged 5–7 Years: The Belfast HAPO Family Study. Bone.

[20] Ahmadieh H., Arabi A. (2011). Vitamins and Bone Health: Beyond Calcium and Vitamin D. Nutr. Rev..

[21] Srour B., Kordahi M.C., Bonazzi E., Deschasaux-Tanguy M., Touvier M., Chassaing B. (2022). Ultra-Processed Foods and Human Health: From Epidemiological Evidence to Mechanistic Insights. Lancet Gastroenterol. Hepatol..

[22] Peters M.D.J., Marnie C., Tricco A.C., Pollock D., Munn Z., Alexander L., McInerney P., Godfrey C.M., Khalil H. (2021). Updated Methodological Guidance for the Conduct of Scoping Reviews. JBI Evid. Implement..

[23] Tricco A.C., Lillie E., Zarin W., O’Brien K.K., Colquhoun H., Levac D., Moher D., Peters M.D.J., Horsley T., Weeks L. (2018). PRISMA Extension for Scoping Reviews (PRISMA-ScR): Checklist and Explanation. Ann. Intern. Med..

[24] Akkaya Z., Joseph G.B., Lynch J.A., McCulloch C., Gassert F., Pedoia V., Sims W., Lane N.E., Link T.M. (2024). The Relationship Between Ultra-Processed Food Intake and Knee Cartilage Thickness in Men and Women:Data From Osteoarthritis Initiative. Osteoarthr. Cartil..

[25] Brooks E.L.G., Tangney C.C., Ritz E.M. (2025). Ultra-Processed Food Intake and Prevalence of Osteoporosis in US Adults Aged 50 Years and Older: A Cross-Sectional Analysis. Osteoporos. Int..

[26] Lim H.-S., Ji S.-I., Hwang H., Kang J., Park Y.-H., Lee H.-H., Kim T.-H. (2018). Relationship between Bone Density, Eating Habit, and Nutritional Intake in College Students. J. Bone Metab..

[27] Mangano K.M., Sahni S., Kiel D.P., Tucker K.L., Dufour A.B., Hannan M.T. (2017). Dietary Protein Is Associated with Musculoskeletal Health Independently of Dietary Pattern: The Framingham Third Generation Study. Am. J. Clin. Nutr..

[28] Monjardino T., Lucas R., Ramos E., Lopes C., Gaio R., Barros H. (2015). Associations between a Posteriori Defined Dietary Patterns and Bone Mineral Density in Adolescents. Eur. J. Nutr..

[29] Nguyen K., Vergne-Salle P., Fressinaud-Marie A.-C., Bertin P. (2019). AB0709 Influence of Dietetary Intakes on Spondyloarthritis: An Observational Prospective Study. Ann. Rheum. Dis..

[30] Noel S., Fouhy L., Flanagan K., Mangano K., Zhang X., Tucker K. (2023). P23-058-23 Ultra-Processed Food Intake and Osteoporosis Among Puerto Rican Adults. Curr. Dev. Nutr..

[31] Saito M., de Oliveira B., Macedo A., dos Santos C., Lopes R., Yamanaka J., Shimano A. (2024). Cafeteria Diet Can Affect Bone Microarchitecture in Sedentary and Trained Male Rats. J. Clin. Densitom..

[32] Shin S., Sung J., Joung H. (2015). A Fruit, Milk and Whole Grain Dietary Pattern Is Positively Associated with Bone Mineral Density in Korean Healthy Adults. Eur. J. Clin. Nutr..

[33] Shin S., Hong K., Kang S.W., Joung H. (2013). A Milk and Cereal Dietary Pattern Is Associated with a Reduced Likelihood of Having a Low Bone Mineral Density of the Lumbar Spine in Korean Adolescents. Nutr. Res..

[34] Sims W., Akkaya Z., Joseph G., Lynch J.A., Gassert F., McCulloch C., Lane N.E., Link T.M. (2024). Association of Ultra-Processed Food Intake and Sex Differences in Osteoarthritis-Related Pain and Clinical Performance. Osteoarthr. Cartil..

[35] Smaira F.I., Mazzolani B.C., Peçanha T., Dos Santos K.M., Rezende D.A.N., Araujo M.E., Bonfiglioli K., Scagliusi F.B., Benatti F.B., De Sá Pinto A.L. (2020). Ultra-Processed Food Consumption Associates with Higher Cardiovascular Risk in Rheumatoid Arthritis. Clin. Rheumatol..

[36] Travinsky-Shmul T., Beresh O., Zaretsky J., Griess-Fishheimer S., Rozner R., Kalev-Altman R., Penn S., Shahar R., Monsonego-Ornan E. (2021). Ultra-Processed Food Impairs Bone Quality, Increases Marrow Adiposity and Alters Gut Microbiome in Mice. Foods.

[37] Vogel C., Parsons C., Godfrey K., Robinson S., Harvey N., Inskip H., Cooper C., Baird J. (2016). Greater Access to Fast-Food Outlets Is Associated with Poorer Bone Health in Young Children. Osteoporos. Int..

[38] Wang S., Xie J., Zhai D., Wang Z., Qi H., Deng M. (2024). Association between Ultra-Processed Food and Osteoporosis: A Cross-Sectional Study Based on the NHANES Database. Nutr. Metab..

[39] Wei Y., Zhang T., Liu Y., Liu H., Zhou Y., Su J., Chen L., Bai L., Xia Y. (2024). Ultra-Processed Food Consumption, Genetic Susceptibility, and the Risk of Hip/Knee Osteoarthritis. Clin. Nutr..

[40] Zaretsky J., Griess-Fishheimer S., Carmi A., Travinsky Shmul T., Ofer L., Sinai T., Penn S., Shahar R., Monsonego-Ornan E. (2021). Ultra-Processed Food Targets Bone Quality via Endochondral Ossification. Bone Res..

[41] Zhang T., Xu X., Chang Q., Lv Y., Zhao Y., Niu K., Chen L., Xia Y. (2024). Ultraprocessed Food Consumption, Genetic Predisposition, and the Risk of Gout: The UK Biobank Study. Rheumatology.

[42] Zhao H., Bai Y., Liu Y., Xing Y., Yan Y., Chen G., Chen J., Wang X., Chen C., Zhang Z. (2024). Association of Ultraprocessed Food Consumption with Risk of Rheumatoid Arthritis: A Retrospective Cohort Study in the UK Biobank. Am. J. Clin. Nutr..

[43] Page M.J., McKenzie J.E., Bossuyt P.M., Boutron I., Hoffmann T.C., Mulrow C.D., Shamseer L., Tetzlaff J.M., Akl E.A., Brennan S.E. (2021). The PRISMA 2020 Statement: An Updated Guideline for Reporting Systematic Reviews. BMJ.

[44] Khoury N., Martínez M.Á., Garcidueñas-Fimbres T.E., Pastor-Villaescusa B., Leis R., De Las Heras-Delgado S., Miguel-Berges M.L., Navas-Carretero S., Portoles O., Pérez-Vega K.A. (2024). Ultraprocessed Food Consumption and Cardiometabolic Risk Factors in Children. JAMA Netw. Open.

[45] Suhett L.G., Filgueiras M.D.S., De Novaes J.F., Sukumar D. (2023). Role of Diet Quality in Bone Health in Children and Adolescents: A Systematic Review. Nutr. Rev..

[46] Biver E., Herrou J., Larid G., Legrand M.A., Gonnelli S., Annweiler C., Chapurlat R., Coxam V., Fardellone P., Thomas T. (2023). Dietary Recommendations in the Prevention and Treatment of Osteoporosis. Jt. Bone Spine.

[47] Calcaterra V., Cena H., Rossi V., Santero S., Bianchi A., Zuccotti G. (2023). Ultra-Processed Food, Reward System and Childhood Obesity. Children.

[48] Khandpur N., Neri D.A., Monteiro C., Mazur A., Frelut M.-L., Boyland E., Weghuber D., Thivel D. (2020). Ultra-Processed Food Consumption among the Paediatric Population: An Overview and Call to Action from the European Childhood Obesity Group. Ann. Nutr. Metab..

[49] Luiten C.M., Steenhuis I.H., Eyles H., Ni Mhurchu C., Waterlander W.E. (2016). Ultra-Processed Foods Have the Worst Nutrient Profile, yet They Are the Most Available Packaged Products in a Sample of New Zealand Supermarkets. Public Health Nutr..

[50] Martínez Leo E.E., Peñafiel A.M., Hernández Escalante V.M., Cabrera Araujo Z.M. (2021). Ultra-Processed Diet, Systemic Oxidative Stress, and Breach of Immunologic Tolerance. Nutrition.

[51] Tristan Asensi M., Napoletano A., Sofi F., Dinu M. (2023). Low-Grade Inflammation and Ultra-Processed Foods Consumption: A Review. Nutrients.

[52] Gioia C., Lucchino B., Tarsitano M.G., Iannuccelli C., Di Franco M. (2020). Dietary Habits and Nutrition in Rheumatoid Arthritis: Can Diet Influence Disease Development and Clinical Manifestations?. Nutrients.

[53] Zhang Y., Chen S., Yuan M., Xu Y., Xu H. (2022). Gout and Diet: A Comprehensive Review of Mechanisms and Management. Nutrients.

[54] Qu Y., Hu W., Huang J., Tan B., Ma F., Xing C., Yuan L. (2024). Ultra-Processed Food Consumption and Risk of Cardiovascular Events: A Systematic Review and Dose-Response Meta-Analysis. eClinicalMedicine.

[55] Qi L. (2009). Mendelian Randomization in Nutritional Epidemiology. Nutr. Rev..

[56] Naska A., Lagiou A., Lagiou P. (2017). Dietary Assessment Methods in Epidemiological Research: Current State of the Art and Future Prospects. F1000Research.

[57] Popkin B.M., Barquera S., Corvalan C., Hofman K.J., Monteiro C., Ng S.W., Swart E.C., Taillie L.S. (2021). Towards Unified and Impactful Policies to Reduce Ultra-Processed Food Consumption and Promote Healthier Eating. Lancet Diabetes Endocrinol..

