# Supplementary material for: Ultra-Processed Food and Its Impact on Bone Health and Joint Diseases: A Scoping Review
Source: Nutrients. 2025 Mar 28;17(7):1188. doi: 10.3390/nu17071188 (PMC11990240; doi:10.3390/nu17071188)
Supplement: Supplementary file 1 [file nutrients-17-01188-s001.zip › Search strategy.pdf]

PubMed search string

("Ultra-Processed Foods"[All Fields] OR "highly processed food"[All Fields] OR "fast food"[All Fields])

AND

("Bone Diseases"[Mesh] OR "Osteoporosis"[Mesh] OR "osteopenia"[All Fields] OR "bone health"[All Fields] OR "bone loss"[All Fields] OR "bone quality"[All Fields] OR "bone density"[Mesh] OR "mineral density"[All Fields] OR "fracture"[All Fields] OR "age-related bone loss"[All Fields] OR "osteosarcopenia"[All Fields] OR "joint diseases"[Mesh] OR "osteoarthritis"[Mesh] OR "rheumatoid arthritis"[All Fields] OR "psoriatic arthritis"[All Fields] OR "spondyloarthritis"[All Fields] OR "ankylosing spondylitis"[All Fields] OR "gout"[All Fields] OR "chronic bone diseases"[All Fields] OR "chronic joint diseases"[All Fields])

Web of Science search string

TS=("Ultra-Processed Foods" OR "highly processed food" OR "fast food")

AND

TS=("Bone Diseases" OR "Osteoporosis" OR "osteopenia" OR "bone health" OR "bone loss" OR "bone quality" OR "bone density" OR "mineral density" OR "fracture" OR "age-related bone loss" OR "osteosarcopenia" OR "joint diseases" OR "osteoarthritis" OR "rheumatoid arthritis" OR "psoriatic arthritis" OR "spondyloarthritis" OR "ankylosing spondylitis" OR "gout" OR "chronic bone diseases" OR "chronic joint diseases")

Embase search string

('ultra-processed food':ti,ab OR 'highly processed food':ti,ab OR 'fast food':ti,ab)

AND

('bone disease':ti,ab OR 'osteoporosis':ti,ab OR 'osteopenia':ti,ab OR 'bone health':ti,ab OR 'bone loss':ti,ab OR 'bone quality':ti,ab OR 'bone density':ti,ab OR 'mineral density':ti,ab OR 'fracture':ti,ab OR 'age-related bone loss':ti,ab OR 'osteosarcopenia':ti,ab OR 'joint disease':ti,ab OR 'osteoarthritis':ti,ab OR 'rheumatoid arthritis':ti,ab OR 'psoriatic arthritis':ti,ab OR 'spondyloarthritis':ti,ab OR 'ankylosing spondylitis':ti,ab OR 'gout':ti,ab OR 'chronic bone disease':ti,ab OR 'chronic joint disease':ti,ab)
